# Advances in Lipid Nanoparticles for siRNA Delivery

**DOI:** 10.3390/pharmaceutics5030498

**Published:** 2013-09-18

**Authors:** Yuen Yi C. Tam, Sam Chen, Pieter R. Cullis

**Affiliations:** Department of Biochemistry and Molecular Biology, University of British Columbia, 2350 Health Sciences Mall, Vancouver, B.C. V6T 1Z3, Canada; E-Mails: s.chen@alumni.ubc.ca (S.C.); pieterc@mail.ubc.ca (P.R.C.)

**Keywords:** lipid nanoparticle, siRNA, ionizable amino lipid, pKa, PEG lipid, targeting

## Abstract

Technological advances in both siRNA (small interfering RNA) and whole genome sequencing have demonstrated great potential in translating genetic information into siRNA-based drugs to halt the synthesis of most disease-causing proteins. Despite its powerful promises as a drug, siRNA requires a sophisticated delivery vehicle because of its rapid degradation in the circulation, inefficient accumulation in target tissues and inability to cross cell membranes to access the cytoplasm where it functions. Lipid nanoparticle (LNP) containing ionizable amino lipids is the leading delivery technology for siRNA, with five products in clinical trials and more in the pipeline. Here, we focus on the technological advances behind these potent systems for siRNA-mediated gene silencing.

## 1. Introduction

RNA interference (RNAi), since its discovery in the 1990s, has rapidly become the most powerful tool for studying functional genomics and validating gene targets *in vitro* and *in vivo*, as well as developing gene-specific medicines [1,2]. The small interfering RNA (siRNA) is a double-stranded oligonucleotide composed of typically 19–25 base pairs. It binds to the RNA-induced silencing complex (RISC) and guides the cleavage of the targeted mRNA sequence. Advances in design algorithms and nucleotide chemistry for siRNA, combined with the completion of the human genome and advances in whole genome sequencing technologies, have presented exciting opportunities to rapidly translate genomic information (such as whole genome sequencing of tumour tissue to identify causal genes) into siRNA-based therapeutics to block the production of disease-causing proteins [2,3,4]. siRNAs can be designed to target most genes in the genome, from one gene at a time to several genes simultaneously, vastly expanding the number of “druggable” targets.

The major limitation of siRNA use in the clinic is the development of safe and effective systemic delivery systems. “Naked” siRNA is highly unstable and rapidly degraded by nucleases in biological fluids, does not accumulate in target tissues and cannot readily cross target cell membranes to access its cytoplasmic site of action [5,6]. Although there are significant advancements in siRNA design and chemical modifications that both increase stability and reduce immunogenicity [7,8,9,10], there is still a need for efficient cellular uptake and target site accumulation. Appropriate delivery vehicles are therefore essential for realizing the potential of siRNA technology. Delivery strategies include both polymer- and lipid-based systems [11,12]. Among all delivery platforms, lipid nanoparticles (LNPs) containing ionizable amino lipids represent the leading system for intravenously (*i.v.*) administered RNAi-based therapeutics. It has been shown that LNP siRNA systems are capable of hepatocyte gene silencing *in vivo* at doses as low as 0.005 mg/kg in animal models following *i.v.* injection, which is currently the worldwide gold standard for siRNA-based therapeutics [13,14]. Five LNP siRNA formulations are currently in various stages of clinical development. Alnylam Pharmaceuticals has three products in their pipeline, which target transthyretin (TTR) for the treatment of TTR-mediated amyloidosis (ALN-TTR02), vascular endothelial growth factor (VEGF) and kinesin spindle protein (KSP) for hepatocellular carcinoma (ALN-VSP), and proprotein convertase subtilisin/kexin type 9 (PCSK9) for hypercholesterolemia (ALN-PCS). In addition, Tekmira Pharmaceuticals has developed TKM-PLK1 that targets polo-like kinase 1 (PLK1) for solid tumors and TKM-Ebola to treat Ebola virus infection. All five products show very safe clinical profiles and promising activity. They are described in detail in other reviews [15,16,17,18,19] and their respective company websites (http://www.alnylam.com/Programs-and-Pipeline/index.php and http://www.tekmirapharm.com/Programs/Products.asp). This review is focused on the technological bases behind these advanced LNP systems.

## 2. LNP Formulation: Composition, Structure and Size

**Figure 1:**
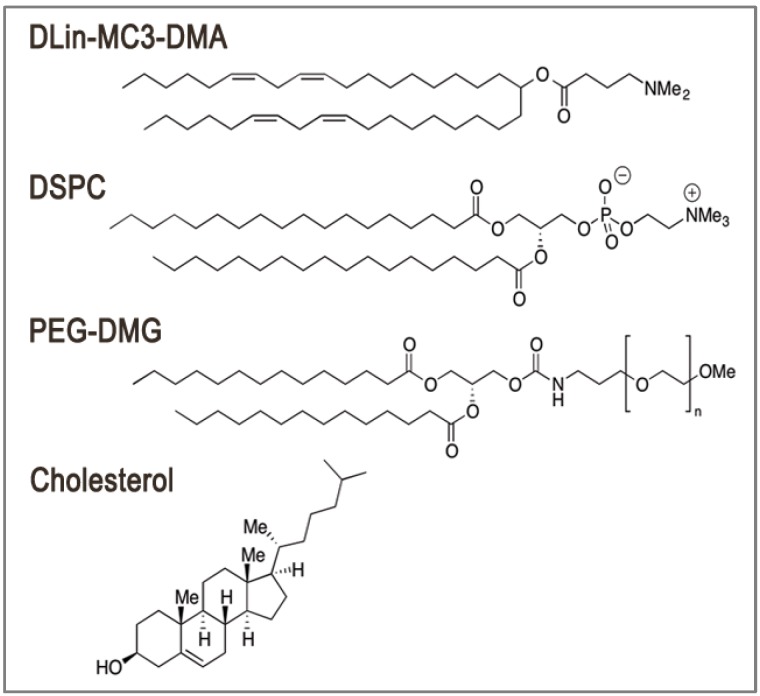
Lipid nanoparticle (LNP) siRNA systems are typically composed of ionizable amino lipids, phosphatidylcholine lipids, cholesterol and polyethylene glycol-lipid conjugate (PEG-lipids). Structure of heptatriaconta-6,9,28,31-tetraen-19-yl 4-(dimethylamino)butanoate (DLin-MC3-DMA), 1,2-distearoyl-*sn*-glycero-3-phosphocholine (DSPC), polyethylene glycol-dimyristolglycerol (PEG-DMG) and cholesterol is shown.

**Figure 2:**
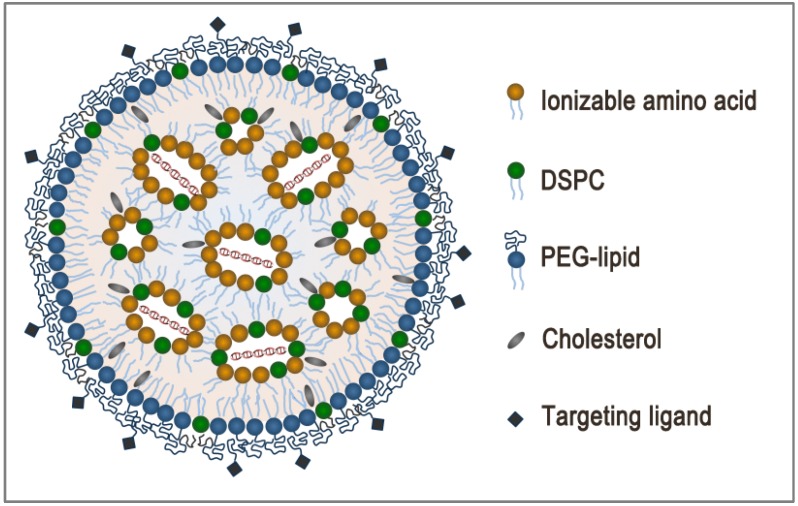
A schematic of LNP siRNA showing a nanostructured core.

The advanced LNP siRNA systems are lipid-based particles with diameters less than 100 nm as measured by dynamic light scattering and cryo-transmission electron microscopy (cryoTEM). They are composed of an ionizable amino lipid (e.g., heptatriaconta-6,9,28,31-tetraen-19-yl 4-(dimethylamino)butanoate, DLin-MC3-DMA [14]), a phosphatidylcholine (1,2-distearoyl-sn-glycero-3-phosphocholine, DSPC), cholesterol and a coat lipid (polyethylene glycol-dimyristolglycerol, PEG-DMG) at molar ratio of 50:10:38.5:1.5 (Figure 1). CryoTEM revealed that LNP systems formed by mixing an ethanol stream containing the lipid mixture with an aqueous stream containing the siRNA, by either a T-tube [20] or microfluidic-based mixing [21,22], have an electron-dense core instead of the less dense aqueous core characteristic of vesicular structures [21,23]. Indeed, the LNP siRNA systems have an interior lipid core containing siRNA complexed with ionizable cationic lipid, as shown by the absence of ^31^P NMR signal from free phosphorothioate in the siRNA and the complete protection of siRNA degradation by external RNases [21]. Computer simulation of the self-assembly of lipids suggests a nanostructured core in which siRNA is located in internal inverted micelles complexed with ionizable amino lipid (Figure 2). Furthermore, DSPC interacts with the siRNA phosphate through its choline group, cholesterol is dispersed about evenly between the core and surface, and the polyethylene glycol-lipid conjugate (PEG-lipid) is distributed predominantly on the surface. It is postulated that the rapid mixing of lipid components with the siRNA allows for the formation of nucleating structures composed of siRNA and ionizable amino lipid. As the polarity of the mixing environment increases, coating of the nucleating structures by the remaining lipids occurs until they reach their solubility limits, thus forming the final LNP siRNA structure [21]. The size of LNP siRNA systems made by the microfluidic technique is dictated by the PEG-lipid content [22]. As the amount of PEG-lipid increases from 0.5 mol% to 5 mol%, LNP diameter decreases from approximately 100 nm to 25 nm. siRNA encapsulation efficiencies are unaffected and remain greater than 95% [21,22].

## 3. Encapsulation and Intracellular Release: Dual Function of Ionizable Amino Lipids

A major advancement in the design of ionizable cationic lipids was the modulation of their apparent acid dissociation constant (pK_a_). pK_a_ values of 7 and lower have been shown to be critical for encapsulation of nucleic acids and *in vivo* activity [24,25]. In environments where the pH is below the pK_a_ of the ionizable lipid (e.g., pH 4.0), the amino group is protonated and interacts with the negatively charged nucleic acids, thereby promoting the self-assembly of the formulation components into nanoparticles encapsulating the siRNA. In physiological environments where the pH is above the pK_a_ of the ionizable lipid (e.g., pH 7.4), the surface of the LNP has an almost neutral charge, which improves circulation and reduces toxicity. Subsequently in the acidic environment of endosomes, the amino group of the ionizable lipid is positively charged and associates with the anionic endosomal lipids. This interaction enables the destabilization of the endosomal membranes and promotes the release of siRNA into the cytosol [26,27]. 

The first ionizable amino lipid that was used for nucleic acid encapsulation was 1,2-dioleoyl-3-dimethyaminopropane (DODAP), which has a pKa of 6.6–7 and one double bond in each of its acyl chains [24,25,28]. Subsequent work focused on the impact of the number of double bonds in the acyl chain demonstrated that ionizable lipids containing fully saturated acyl chains showed no silencing of luciferase activity *in vitro*, whereas ionizable lipids containing two or three double bonds per acyl chain showed enhanced silencing activity [29]. Encapsulation efficiencies of siRNA seemed to be compromised using ionizable lipids that contain three double bonds per acyl chain. Therefore, the linoleyl lipid has become the acyl chain of choice in subsequent ionizable lipid development. 

A structure-activity relationship guided synthesis and screening of a large number of ionizable lipids with various types of linkers connecting the amino group and the acyl chains. These studies have identified DLin-KC2-DMA (2,2-dilinoleyl-4-(2-dimethylaminoethyl)-[1,3]-dioxolane) [13], and DLin-MC3-DMA [14], which are 100-fold and 1000-fold more potent, respectively, in silencing of a hepatic gene (Factor VII) in comparison to the previous generation lipid DLin-DMA (1,2-dilinoleyloxy-*N*,*N*-dimethyl-3-aminopropane) [29]. The ED_50_ (median effective dose) for LNP containing DLin-MC3-DMA to silence Factor VII in mice and TTR in non-human primates was 0.005 mg/kg and 0.03 mg/kg, respectively [14]. One of the key findings from these studies was the optimum lipid pK_a_ value of 6.2–6.5 as a dominating factor in determining hepatic gene-silencing activity *in vivo*. DLin-MC3-DMA, having a pKa of 6.44, is currently the most active ionizable lipid being used in clinical trials. 

In hopes of further promoting biocompatibility, a novel generation of ionizable amino lipids was recently synthesized with biodegradable ester functionalities in the acyl chains [30]. These novel lipids were well tolerated by the animals and rapidly eliminated from plasma and tissues. Importantly, they exhibited excellent potencies (ED_50_ < 0.01 mg/kg in mice, similar to that of DLin-MC3-DMA) in both rodent and non-human primate models. Although the relevance of these findings to human treatment remains to be proven, these preclinical results for biodegradable LNP siRNA formulations show great promise for clinical applications.

## 4. Steric Barrier: Stability and Circulation Lifetime

Unprotected lipid-based delivery systems are rapidly cleared by the mononuclear phagocyte system [31,32]. In order to overcome this, hydrophilic polyethylene glycol has been widely used to coat lipid-based delivery systems, which prevents aggregation during the formulation process as well as provide “stealth” like characteristics post *i.v.* administration. The optimized PEG-lipid characteristics in current LNP siRNA systems were largely derived from earlier work on PEGylated liposomes [33,34,35] and stabilized plasmid or antisense lipid particles (SPLP or SALP). It was found that SPLP or SALP with PEG-ceramide that have C_20_ anchors (PEG-CerC_20_) exhibited poor transfection efficiency in cultured cells compared to its shorter anchor counterparts such as PEG-CerC_14_ or PEG-CerC_8_ [36,37,38]. The length of the ceramide lipid anchor dictated how long the PEG-lipid remained associated with the LNP. PEG-lipids with longer acyl anchors remain associated with the LNP longer, thus preventing LNP interaction with cells and subsequently with the endosomal membranes. They also provide the LNP with a more durable coat, which extends circulation lifetime [39,40]. Consistent findings were observed when PEG-ceramides were replaced with PEG-succinoyl-diacylglycerols (PEG-s-DAG) that have various acyl anchor lengths [41]. Due to the ease of synthesis and purification, PEG-s-DAG replaced PEG-Cer in later SPLP formulations and long acyl chain PEG-s-DAG such as PEG-s-distearoylglycerol (PEG-s-DSG) were used to increase circulation lifetime in the hope of exploiting the enhanced permeability and retention effect at tumor sites.

Unfortunately, despite being efficacious and non-toxic after a single bolus injection, PEGylated LNP with long acyl anchors (PEG-DSPE (1,2-distearoyl-*sn*-glycero-3-phosphoethanolamine), PEG-s-DSG, or PEG-CerC_20_) were rapidly cleared upon repeated administration as a result of a robust immune response to the PEG component [42,43]. The use of rapidly dissociated shorter anchor PEG-lipids such as PEG-s-DMG or PEG-CerC_14_ however, mitigated this immunogenic response. Coincidentally, it was also observed that LNP containing PEG-s-DAG progressively lost the PEG moiety due to its succinate linker leading to particle aggregation and a reduced shelf-life. In response to this, the succinate linker was replaced with a carbamate linker to confer improved chemical stability without affecting efficacy [44].

## 5. Cellular Uptake: Endogenous and Exogenous Targeting Ligands

Neutral liposomes have been shown to bind to proteins in serum, exchange components with lipoproteins and acquire factors that can potentially target them to specific cell types [45,46]. In particular, they interact with apolipoprotein (Apo) E and A-I [47,48]. ApoE, but not ApoA-I or ApoA-IV, was further found to enhance uptake of neutral liposomes in HepG2 cells and primary hepatocytes [49]. The role of ApoE in LNP uptake into hepatocytes was confirmed *in vivo* using ApoE-deficient mice [50]. The authors demonstrated that LNP were cleared more slowly from the circulation and were taken up by hepatocytes at least 20-fold less in ApoE-deficient mice than in wild-type animals. Similarly, LNP siRNA systems containing ionizable lipids require ApoE for activity [51]. Silencing of Factor VII was compromised in mice lacking ApoE or low-density lipoprotein (LDL) receptor, suggesting that ApoE acts as an endogenous ligand for LNP siRNA systems that facilitate uptake into hepatocytes via the LDL receptor. 

When endogenous ligands are not available, exogenous ligands can be used to enhance uptake of LNP siRNA systems in target cells. Exogenous ligands such as antibodies, antibody fragments and peptides have been widely used in the field of liposome technology [52,53,54,55]. However, they are expensive and difficult to manufacture, as well as potentially immunogenic. In contrast, small molecule ligands conjugated to the distal end of PEG-lipids are simple to synthesize and can be formulated into LNP in a straightforward manner (Figure 2) [51,56,57]. *N*-Acetylgalactosamine (GalNAc), which binds with high affinity to the asialoglycoprotein receptor (ASGPR) found on hepatocytes, have been shown to rescue gene-silencing activity of LNP siRNA systems in ApoE deficient mice [51]. Other exogenous small molecule ligands have shown utility in non-hepatic cells. Anisamide, which interacts with sigma receptors, increases delivery and activity of siRNA containing nanoparticles in lung tumors and metastases [56,58,59]. Furthermore, strophanthidin, a cardiac glycoside that binds to the ubiquitously expressed cell surface receptor Na^+^/K^+^ ATPase, has been shown to enhance delivery of LNP siRNA to various cell types originating from the ovary, breast, pancreas, lung and prostate [57]. While the delivery of LNP using a promiscuous ligand may be non-specific, target specificity can be conferred using siRNA against disease-causing genes that are expressed in specific tissues.

## 6. Concluding Remarks

Using LNP as the delivery platform for siRNA has undoubtedly increased the potential of siRNA as therapeutics. This is evidenced by five LNP siRNA formulations for various liver-related diseases in clinical trials and more in the pipeline. The increased understanding of individual formulation components and their composition, LNP structure as well as formulation methods have led to the development of increasingly potent LNP siRNA systems. The success of LNP in hepatic applications is due, at least in part, to the liver’s ideal physiology, notably being highly perfused with fenestrated endothelium. Other contributing factors include the optimum pK_a_ of the ionizable lipid, dissociable PEG-lipid, and association of LNP with endogenous ligand ApoE.

A number of challenges remain to be overcome for LNP siRNA to be used as therapeutics in a broad range of diseases. The existing LNP siRNA systems for liver applications have to be modified to extend their utility for non-hepatic tissues such as distal tumors. Novel small molecule targeting ligands will be required to facilitate LNP uptake into these non-hepatic tissues, especially when the siRNA is not tissue-specific. In order to reach tumor cores or tumors with poor vascularization, small LNP may be useful [60,61]. LNP siRNA systems as small as 25 nm can be made using microfluidic micromixing technology [22]; however, the relatively small siRNA payload may compromise activity and alternative methods to increase potency will have to be explored. Furthermore, LNP composition will likely require modifications for other routes of administration such as intraperitoneal, subcutaneous, intranasal or topical.
